# Heart Failure Re-Admission: Measuring the Ever Shortening Gap between Repeat Heart Failure Hospitalizations

**DOI:** 10.1371/journal.pone.0106494

**Published:** 2014-09-11

**Authors:** Jeffrey A. Bakal, Finlay A. McAlister, Wei Liu, Justin A. Ezekowitz

**Affiliations:** 1 Patient Health Outcomes Research and Clinical Effectiveness Unit, University of Alberta, Edmonton, Alberta, Canada; 2 Division of General Internal Medicine, University of Alberta, Edmonton, Alberta, Canada; 3 Division of Cardiology, University of Alberta, Edmonton, Alberta, Canada; University Hospital of Würzburg, Germany

## Abstract

Many quality-of-care and risk prediction metrics rely on time to first rehospitalization even though heart failure (HF) patients may undergo several repeat hospitalizations. The aim of this study is to compare repeat hospitalization models. Using a population-based cohort of 40,667 patients, we examined both HF and all cause re-hospitalizations using up to five years of follow-up. Two models were examined: the gap-time model which estimates the adjusted time between hospitalizations and a multistate model which considered patients to be in one of four states; community-dwelling, in hospital for HF, in hospital for any reason, or dead. The transition probabilities and times were then modeled using patient characteristics and number of repeat hospitalizations. We found that during the five years of follow-up roughly half of the patients returned for a subsequent hospitalization for each repeat hospitalization. Additionally, we noted that the unadjusted time between hospitalizations was reduced ∼40% between each successive hospitalization. After adjustment each additional hospitalization was associated with a 28 day (95% CI: 22-35) reduction in time spent out of hospital. A similar pattern was seen when considering the four state model. A large proportion of patients had multiple repeat hospitalizations. Extending the gap between hospitalizations should be an important goal of treatment evaluation.

## Introduction

Heart Failure (HF) is a common disease with a high morbidity and mortality. Each year in the United States, 5.1 million individuals with heart failure undergo ∼1 million hospitalizations, 668,000 emergency department visits and ∼3 million physician outpatient visits resulting in an overall cost of approximately $40 billion per year [1]. Given this public health issue, understanding the patterns of recurrent hospitalizations related to HF is important in order to design therapy or management strategies that can help reduce this burden for individual patients and the system.

The 30 day rehospitalization rates for patients with HF remain high in clinical trials (6.0%) [2], registries (24.5%) and in population health studies (18%) [3]. However, the modeling of rehospitalization remains challenging even using models incorporating a large number of patient characteristics [4]. Most analyses attempting to model re-hospitalizations utilize only the first re-hospitalization and fail to incorporate for the repetitive nature or trajectory of re–hospitalizations in patients with HF. A recent study in Olmstead county showed that while 83% of HF patients were hospitalized at least once, 43% were hospitalized four or more times within five years of diagnosis [5]. Several repeated events methods have been considered for these data and have seen limited uptake in part due to the assumptions required for each model [6].

The temporal trends in these data have been explored previously [7] however to this point in time, few studies examining rehospitalization risk have explored the amount of time between hospitalizations (the “gap-time”) [8], which may play a role in identifying patients at increased risk for morbid events. Conceivably, while two patients may exhibit a similar risk of at least one hospitalization, a sicker patient may be re-admitted to hospital more than once and/or with a shorter duration between hospitalizations.

The objective of this study was to describe the gap-time and transitions in a cohort of patients who were hospitalized more than once with heart failure, and explore the patient and system level features associated with the gap-time. We developed a model to examine the timing and risk of both HF-specific and all-cause hospitalization while simultaneously considering the competing risks of all-cause hospitalization and mortality in HF patients, allowing combined modeling of parameters. An improved understanding of the trajectory of heart failure patients is important for determining changes in health state and planning resources.

## Methods

### Ethics statement

The study was approved by the Health Research Ethics Board at the University of Alberta. Patient consent was not obtained; therefore patient records/information were anonymized and de-identified prior to analysis.

### Databases

The province of Alberta has a single payer, government funded health care system, Alberta Health that provides universal access to its 3.7 million residents. Using the data linked from four administrative databases, a cohort was created that identified patients by identifying their first visit with an inpatient diagnosis of heart failure between January 1, 1999, and December 1, 2008.

The four linked databases include: i) the Discharge Abstract Database (DAD), which records dates for all admissions to acute care facilities with the most responsible and up to 25 other diagnoses or comorbidities; ii) the Ambulatory Care Database (which tracks visits to the Emergency Departments and hospital-based clinics and allows for coding of up to 10 conditions; iii) the physician claims database (which tracks all physician claims for outpatient services and includes up to three diagnoses per encounter); and iv) the registry which contains the birth, death and immigration information for all Alberta residents.

### Hospitalizations

HF hospitalizations were identified as those hospitalizations with a main diagnosis of HF using ICD-9-CM code 428.x (1999–2002) and ICD-10 code I50.x (2002–2008). The accuracy of these ICD codes for HF has been previously validated against chart audit in Alberta and elsewhere [9]–[11]. Other (non-HF) hospitalizations were those associated with any acute hospitalization for any other main diagnosis. Due to differences in care patterns for patients with hospitalizations in non-acute facilities, patients entering from or discharged to long term care were censored and removed from further analysis.

The cohort was separated into three groups: patients with a single HF hospitalization; patients with two HF hospitalizations in the five years of follow-up; and patients with three or more HF hospitalizations. Patients were followed for up to five years from their index visit (median: 952 days; IQR: 188–1826).

### Statistical analysis

Patient characteristics are presented as numbers and percentages for categorical variables and means and standard deviations or medians and IQRs where relevant. Baseline characteristics were compared according to those with a single hospitalization against those with two and three or more hospitalizations.

Comorbidities were evaluated at every hospitalization so that as patients moved through the maximum five years of follow-up, new diagnoses recorded in hospital, an outpatient clinic or an emergency department could be added. Additionally, at each transition outlined below, the total number of emergency department visits where patients were directly discharged without admission (“treat and release”) and physician office visits were counted.

### Multivariable models

**Figure 1:**
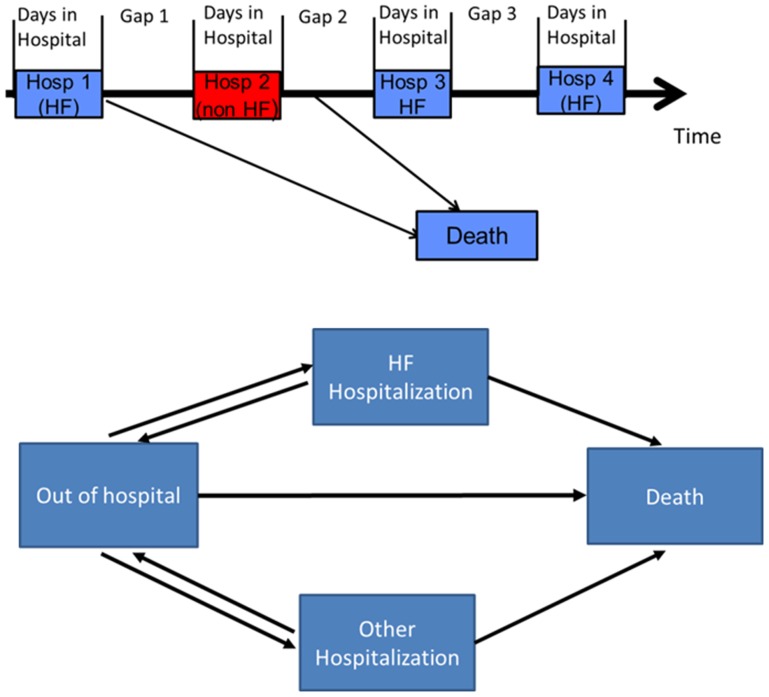
Schematic of timeline for HF patients indicating hospitalizations and Gap times (a); States and transitions for multistate model. Arrows indicate modeled transformations(b).

A series of models were generated adjusted for age, sex, length of stay, acuity of admission, Charlson index, which provides a weighted score based on the number and type of comorbidities, and number of emergency department visits in the six months prior, consistent with the LACE index, which includes the length of stay at the last hospitalization(L), the acuity of the admission (A), the patient's Charlson score (C), and the number of Emergency Department visits in the last six months (E) [12]. Patients were followed for up to five years from their initial HF hospitalization (Figure 1a) to explore changes in gap times as well as the length of stay for each hospitalization. The first set of models utilized a Generalized Estimating Equation (GEE) [8], [13] for the time between hospitalizations. The standard GEE framework examines a single gap-time without consideration for censoring. As such, in the GEE model, we considered the gap-time to be the number of days between discharge from one HF hospitalization until the start of the next hospitalization. Using the GEE approach suggest by Clement, there are limited assumptions about the total number and distribution of events. The GEE model provides a baseline for subsequent model development.

The second model used was a multi-state model [14], [15] where patients are assumed to be in one of four states: 1) Community dwelling (i.e., out of hospital); 2) In hospital with HF as a main diagnosis; 3) In hospital for another reason; or 4) Dead. A multistate model was developed based on both the transitions and time spent in each state; the seven permitted transitions are depicted in Figure 1b. This model considers each non-absorbing state (1 to 3) as a competing risk regression on each available transition, conditional on the patients who have arrived at that state. For example a patient not in hospital is eligible to be hospitalized with HF, hospitalized for another reason, or die as available transitions. This type of model allows for the adjustment of patient factors comorbidities and repeat hospitalizations with a primary diagnosis of HF conditional on the current state. The adjusted times estimates are calculated by comparing the fitted sojourn time (time spent in a given state) between the indicated levels of the target covariate with the other covariates set at the mean level to give an estimate of the effect of changing each variable individually. The index hospitalization is considered to be their first hospitalization with a most responsible diagnosis of HF.

The hospitalizations are considered as episodes of care so that if there are multiple hospitalization records within 24 hours they are treated as a single episode of care. We categorized an episode that had at least one admission with HF as the most responsible diagnosis as an “HF hospitalization” (HFH) while any episode with another main diagnosis as an “Other hospitalization”. The model estimates a fitted hazard ratio for the transitions based on the number of hospitalizations, comorbid conditions and outpatient clinic, physician office and emergency department use in the 180 days prior to the hospitalization. From this model, the sojourn time, i.e., the time spent in a given state, was estimated. Two variants of this model are considered: 1) include the number of times that a patient had a HFH to assess the adjusted change in time for repeat hospitalizations; and 2) exclude this information to account only for independent predictors of transition. As a sensitivity analysis we consider the days alive and out of hospital in the year following the discharge from the index episode. In this model we examine the number of days each patient is both alive and not in acute care as a continuous variable during the subsequent year in a multivariable linear regression framework [2].

All Analyses were complete in SAS v. 9.4 (Cary, NC) and R 2.15.2 (Vienna, Austria) using the “msm” package [14].

## Results

**Figure 2:**
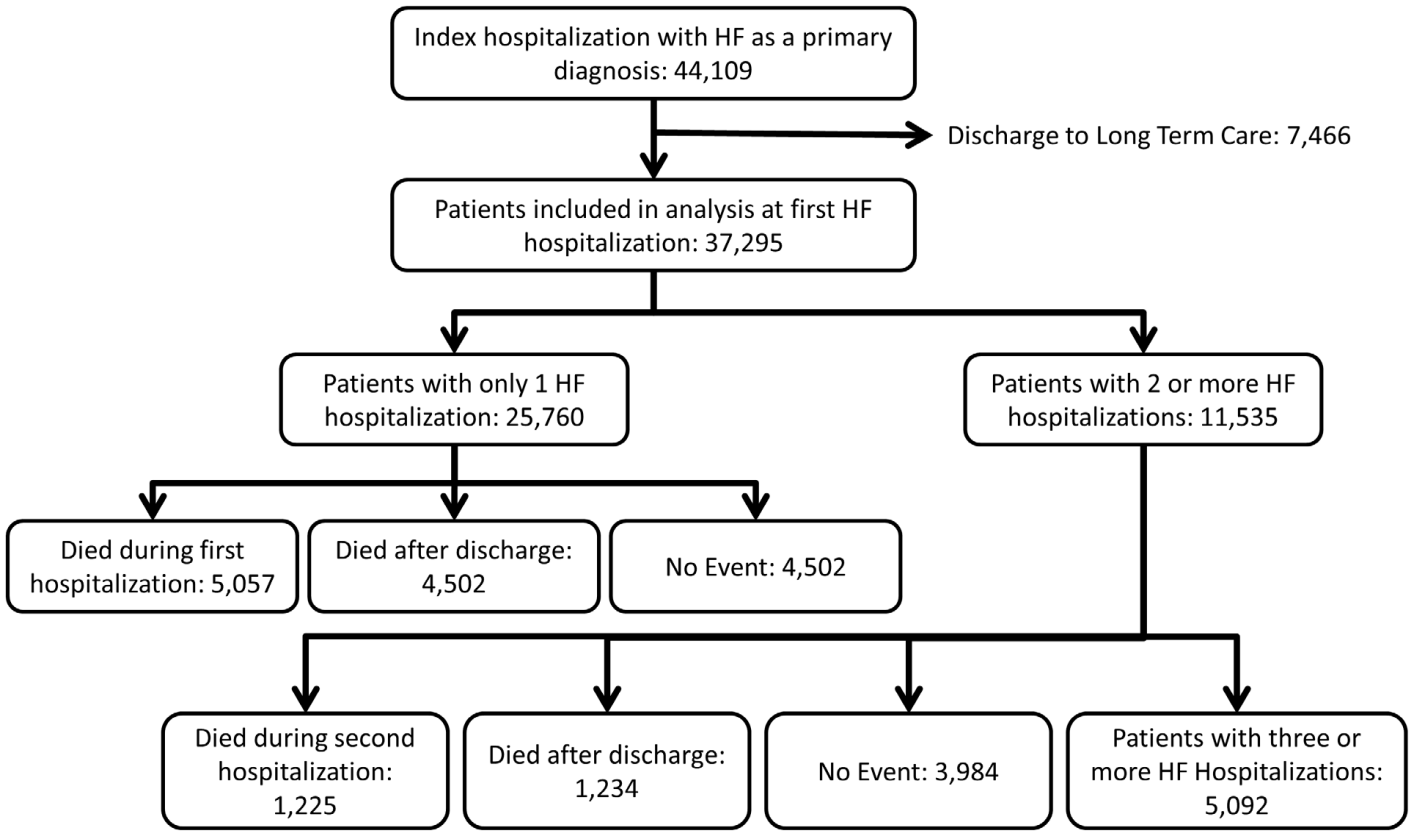
Cohort derivation chart. First two hospitalizations shown

We identified 40,667 patients with a first HFH between January 1, 1999 and December 1, 2008 who were not discharged to long term care (Figure 2). During five years of follow-up, this cohort accounted for 127,927 emergency department visits with discharge (“treat and release” visits), 93,924 total hospitalizations, 794,004 total days in hospital, 59,591 HFH and 14,235 deaths (of which 53% [n = 7,590] occurred during a HF hospitalization. Nearly half of the cohort had no further HFH (27,106) during the median follow-up of 2.6 years. The cohort was split into three groups based on whether or not they had one, two or ≥ three HF hospitalizations. Patients hospitalized more often for HF were older and were more likely to have virtually all comorbidities such as diabetes, peripheral arterial disease and COPD but not dementia or cancer (Table 1).

**Table 1:** Baseline Cohort Demographics.

Variable	Patients with only one HF Hospitalization	Patients with two HF Hospitalizations	Patients with three or more HF Hospitalizations	*p-value*
N (%)	25760 (69.1)	6443 (17.3)	5092 (13.7)	
Median Follow-up days (IQR)	766 (69,1826)	1117 (391,1826)	1368 (696, 1826)	<0.0001
Median Follow-up days: survivors (IQR)	1580 (679, 1826)	1826 (1004, 1826)	1826 (1391, 1826)	<0.0001
Male	13763 (53.4)	3426 (53.2)	2704 (53.1)	0.88
Age mean (SD)	73.4 (13.6)	74.9 (12.5)	75.4 (11.6)	<0.0001
Ischemic Heart disease	14639 (56.8)	3720 (57.7)	3137 (61.6)	<0.0001
Hypertension	16057 (62.3)	4154 (64.5)	3378 (66.3)	<0.0001
Cerebrovascular disease	4734 (18.4)	1179 (18.3)	930 (18.3)	0.98
Atrial fibrillation	8135 (31.6)	2309 (35.8)	1903 (37.4)	<0.0001
Diabetes	7446 (28.9)	2066 (32.1)	1865 (36.6)	<0.0001
Peripheral Vascular disease	3781 (14.7)	1023 (15.88)	893 (17.5)	<0.0001
Renal disease	3638 (14.1)	966 (15.0)	712 (14.0)	0.17
Chronic Obstructive Pulmonary disease	10182 (39.5)	2866 (44.5)	2432 (47.8)	<0.0001
Dementia	2334 (9.1)	415 (7.0)	232 (4.6)	<0.0001
Cancer	4330 (16.8)	916 (14.2)	671 (13.2)	<0.0001
Charlson Score, mean (SD)	4.88 (2.5)	4.77 (2.3)	4.84 (2.2)	0.004
Visits to emergency department during previous 6 months (mean, SD)	0.74 (1.9)	0.83 (1.9)	1.00 (2.6)	<0.0001
Length of Stay at index hospitalization (mean, SD)	10.2 (7.3)	9.7 (6.9)	9.3 (6.6)	<0.0001
LACE score (mean, SD)	12.7 (2.4)	12.7 (2.4)	12.8 (2.3)	0.0002

The models allowed patient comorbidities to change at later visits, but this table provides only the values from the first HF hospitalization.

**Figure 3:**
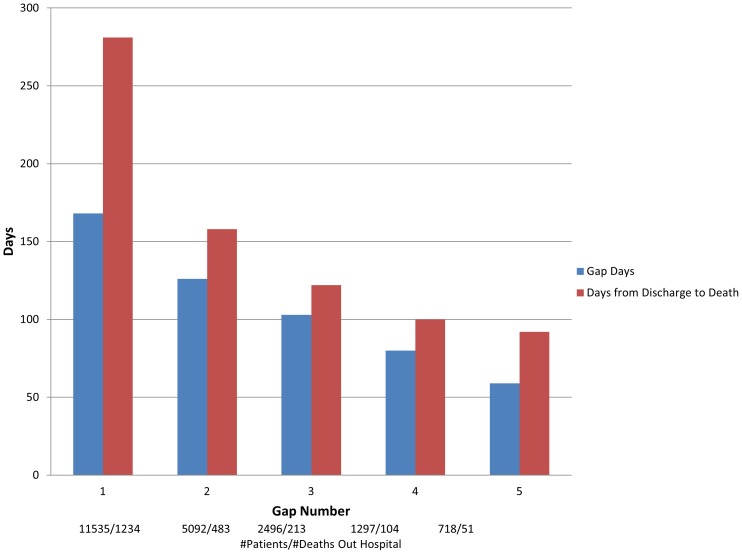
Unadjusted mean times between HF hospitalizations and times to death for HF patients with at least two hospitalizations (1 gap).

**Figure 4:**
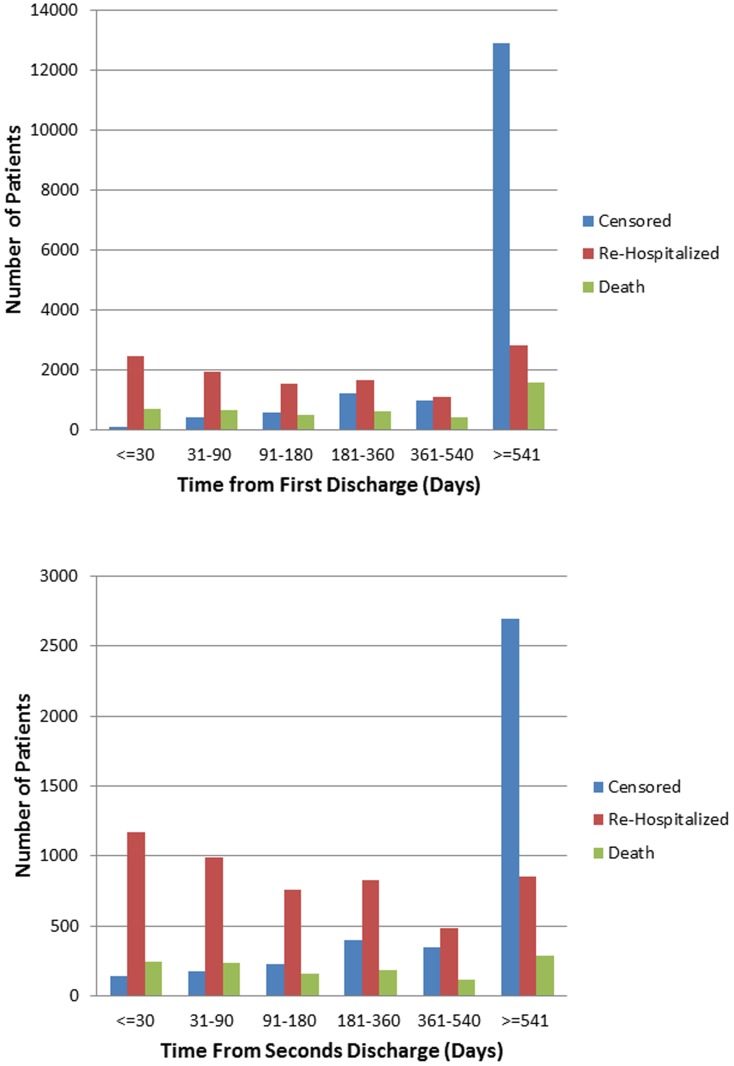
Distribution and times of patients next event following first hospitalization (a); Distribution and times of patients next event following second hospitalization(b).

Although approximately half of the patients were not re-hospitalized for HF after each subsequent hospitalization, as the number of successive HF hospitalizations increased, the number of days between both HF specific and all cause hospitalizations dropped (Figure 3, Table 2). The median and IQR days between the first and second hospitalization for patients was 168 (41–533) and then 126 (35–378), 103 (28–280), 80 (23–235) for the 2^nd^ 3^rd^ and fourth gap between hospitalizations. The distribution of patient events (censored alive, re-hospitalized or died) by time groups are given in Figure 4.

**Table 2:** Adjusted ‘Gap-time’ between hospitalizations in days in GEE model.

Variable	Adjusted time to first event	Adjusted Change in Number of Days between HF hospitalizations	Adjusted Change in Number of Days between all cause hospitalizations	Adjusted Change in Days Alive and out of hospital in the year following index admission
Gap Number	NA	−28.61 (−34.81, −22.41)	−13.62 (−15.39, −11.86)	NA
Male	−0.24 (−10.87, 10.39)	−5.75 (−15.26, −3.76)	2.94 (−2.71, 8.60)	−7.89 (−10.64, −5.14)
Age (per additional year)	−1.98 (−2.40, −1.56)	−2.05 (−2.48, −1.62)	−1.69 (−1.94, −1.44)	−1.90 (−2.00, −1.79)
Length of Stay (per day)	−1.09 (−1.85, −0.33)	−1.13 (−1.85, −0.41)	−0.41 (−0.82, 0.01)	NA
Charlson Score	−18.60 (−20.91, −16.29)	−13.15 (−15.10, −11.20)	−14.62 (−15.69, −13.55)	−13.62 (−14.19, −13.05)
ED visits 180 days prior	−15.99 (−18.18, −13.80)	−8.60 (−10.85, −6.34)	−9.27 (−11.15, −7.38)	−0.97 (−1.39, −0.04)

Negative numbers indicate fewer days between hospitalizations. For example, for any given patient, for each additional year of age, there will be 1.98 fewer days between hospitalizations.

For each additional point on the Charlson score at baseline, patients exhibited 13.2 (95% CI: 11.2 to 15.1) fewer days between HF hospitalizations and 14.6 (95% CI: 13.6 to 15.7) fewer days between any hospitalizations (Table 2). Similarly, for each additional year of age there were 2.1 (95% CI: 1.6 to 2.5) fewer days between HF hospitalizations and 1.7 (95% CI: 1.4 to 1.9) fewer days between any hospitalizations. The time between HF hospitalizations was on average reduced by approximately one month (mean 28.6 days; 95%CI 22.4 to 34.8) for each subsequent HF hospitalization. With the exception of gender, the direction and relative magnitude of each covariate was similar between HF and non HF hospitalizations. As a sensitivity analysis, we explored adjusted days alive and out of hospital in the year following the index HF hospitalization and found that the relevant variables in Table 2 exhibited similar associations with this outcome (although length of stay could not be included as it was in the adjusted model; data available on request). We found that the time to first event analysis and the gap time models display similar coefficients; however we note that the precision and thus statistical significance of each parameter is less in the time to first event model. The gap time and the days alive and out of hospital model were similar with the exception of the impact of number of visits to the Emergency Department in the six months prior. This difference is likely due to the insensitivity of the days alive and out of hospital model to differentiate between long hospitalizations, multiple short hospitalizations and deaths. As a sensitivity analysis we re-examined the data using only patients who were alive at the end of the follow-up time and the results were similar (data available on request).

The competing risk hazards for each transition in the multi-state model are presented in Table 3. For example, for each additional point on the Charlson scale, a patient currently out of hospital has a 4% (95% CI 4% to 5%) higher risk of another HF hospitalization, an 7% higher risk (95%CI 7% to 8%) of being hospitalized for another reason, and a 10% (95%CI 8% to 11%) increased risk of out of hospital death. If the number of prior HF hospitalizations is also considered as a factor in the model, a patient with two prior HFH currently out of hospital has a 28% (95% CI 23% to 33%) higher risk of another HF hospitalization, a 4% higher risk (95%CI 1% to 7%) of being hospitalized for another reason, and a 23% (95%CI 13% to 34%) increased risk if they have already had 3 or more HF hospitalizations.

**Table 3:** Hazard Rates for Multistate models.

	Transition				
	**Out of Hospital to Heart Failure Hospitalization**	**Out of Hospital to Other Hospitalization**	**Death in Hospital Heart Failure Hospitalization**	**Death in Hospital Other Hospitalization**	**Death out of Hospital**
Including Number of Prior HFH					
2 HF Hosp vs 1	1.28 (1.23, 1.33)	1.04 (1.01, 1.07)	0.80 (0.75, 0.85)	0.36 (0.32, 0.42)	0.96 (0.89, 1.03)
3 or more HF Hosp vs 1	3.32 (3.21, 3.43)	1.13 (1.10, 1.17)	0.72 (0.67, 0.78)	1.37 (1.25, 1.51)	1.23 (1.13, 1.34)
Age/5 year	1.03 (1.02, 1.03)	1.03 (1.02, 1.03)	1.09 (1.07, 1.10)	1.00 (0.99, 1.02)	1.15 (1.14, 1.17)
Male vs Female	1.05 (1.02, 1.08)	1.05 (1.02, 1.08)	1.12 (1.06, 1.17)	1.39 (1.30, 1.50)	1.02 (0.96, 1.08)
Number of ED visits 6 months prior	1.00 (0.99, 1.00)	1.00 (0.99, 1.00)	0.99 (0.98, 1.00)	0.95 (0.94, 0.97)	0.95 (0.93, 0.97)
Charlson score	1.04 (1.04, 1.05)	1.07 (1.07, 1.07)	1.05 (1.05, 1.06)	1.06 (1.05, 1.08)	1.10 (1.08, 1.11)
	**Out of Hospital to Heart Failure Hospitalization**	**Out of Hospital to Other Hospitalization**	**Death in Hospital Heart Failure Hospitalization**	**Death in Hospital Other Hospitalization**	**Death out of Hospital**
Excluding number of Prior HFH					
Age/5 year	1.02 (1.02, 1.03)	1.00 (1.00, 1.01)	1.08 (1.07, 1.09)	1.16 (1.15, 1.18)	1.13 (1.12, 1.15)
Male vs Female	1.04 (1.01, 1.06)	0.97 (0.95, 0.99)	1.11 (1.06, 1.15)	0.94 (0.88, 1.01)	0.98 (0.92, 1.04)
Number of ED visits 6 months prior	0.99 (0.99, 1.00)	1.03 (1.02, 1.03)	0.93 (0.92, 0.95)	1.03 (1.03, 1.04)	1.00 (0.99, 1.02)
Charlson Score	1.04 (1.04, 1.05)	1.06 (1.05, 1.06)	1.11 (1.10, 1.12)	1.01 (1.00, 1.03)	1.12 (1.11, 1.14)

Values are hazard rates (95% confidence intervals).

The multistate model extends the days alive and out of hospital model as it allows the estimation of the covariate adjusted time spent in each state (Table 4), as there is no pre-specified or fixed amount of follow-up time. For example, the LOS gives the expected length of stay for a given hospitalization for a patient with the specified demographic/comorbidity level. Each row in the table gives the estimate for that covariate with the other covariates set at their mean values. For example, males spend approximately 5% less time out of hospital despite the fact their length of stay is 10% shorter than female patients (Table 4) – this is because they are more likely to have repeat hospitalizations (Table 3). The time spent out of hospital is reduced with increasing comorbidity load. Increasing age did not appear to affect the time spent in hospital with HF.

**Table 4:** Expected Changes in Adjusted times spent in each state prior to transition with all other covariates set to the mean level.

Comparison	Alive and out of Hospital Mean Change in Days (95%CI)	Length of stay HF Hospitalization Mean Change in Days (95%CI)	Length of stay Non HF Hospitalization Mean Change in Days (95%CI)
Two HF Hospitalizations vs one	−25.47 (−35.22, −16.40)	0.13 (−0.17, 0.42)	−0.21 (−0.44, 0.01)
Three or more HF Hospitalizations vs 2	−94.52 (−97.26, −91.46)	−0.17 (−0.46, 0.13)	−0.29 (−0.48, −0.09)
80 years of age vs 70	−2.88 (−2.89, −2.87)	0.10 (0.09, 0.10)	0.30 (0.29, 0.31)
Male vs Female	2.17 (2.05, 2.29)	0.08 (0.07, 0.09)	0.34 (0.32, 0.36)
1 ED visit in the prior 6 months vs none	−2.06 (−2.17, −1.95)	−0.10 (−0.10, −0.09)	0.09 (0.08, 0.10)
1 point above average on Charlson score	−15.71 (−16.58, −14.88)	0.09 (0.08, 0.10)	0.17 (0.16, 0.18)

## Discussion

Our study demonstrates that the gap-time decreases between each successive hospitalization and this pattern is consistent in both models after adjusting for key prognostic variables such as age, number of Emergency Department visits, and Charlson score. Reporting gap times represent an alternative means of understanding the patient experience that traditional outcomes such as time to first event or proportion of patients who have that event in a defined time frame cannot.

There are a variety of other proportional hazards based repeated events models that have been proposed in the literature [6], these models have the advantage of providing additional information with respect to the models. The Andersen-Gill modifies the proportional hazards model by allowing patients to remain a risk even after the event has occurred, and assumes all events are equal. Other formulations consider stratification and ordering of the events, and do not directly model the time between events. There are important considerations in these models in terms of understanding if there are limits on the number of events and the distribution expected time between events, as well as how to consider the end of follow-up [13].

The use of gap time for evaluating the impact of interventions allows insight beyond simple proportion of patients with mortality/re-hospitalization (after all, if follow-up is sufficiently long then this rate approaches 100% for all patients). This is possible by first separating the re-hospitalizations from the mortality events, then separating the HF hospitalizations from the other hospitalizations, and finally considering both the changing risk and the time spent in the community between multiple hospitalizations. Future studies can utilize this framework to increase the information included in the models. Indeed, there are increasing calls to consider multiple events when evaluating interventions for HF patients but it is important to remember that improved survival outcomes may create multiple re-hospitalizations [16], [17].

The gap time concept is similar in concept to the “days alive and out of hospital” which has been reported in several clinical trials [2], [18], [19]. As an outcome measurement, “days alive and out of hospital” has the advantage of being a simple metric, but it cannot provide information on how the time was distributed *between* hospitalizations nor does it differentiate if an intervention reduced in-hospital time, death, or both.

There have been numerous attempts to develop prediction scores for HF patients (CMS, LACE etc.) [4], [20], [21]. These models, typically based on 30 day outcomes have not been highly predictive with c-indices between 0.5 and 0.7 [4]. There are three principal reasons that these models do not achieve higher predictive accuracy. First, some (e.g., LACE, LaCE) combine the death and rehospitalization endpoints resulting in a lack of differentiation between those risk factors related to dying and those associated with being re-hospitalized. Secondly, 30 day follow-up windows have no direct physiological implication (i.e., a patient having an event on day 31 is likely similar to one having an event on day 30). Third, these scores typically predict time to first event rather than consideration of total number of events over a longer duration of time. The gap-time method allows the incorporation of the factors related to the multiple events, and additionally provides an indication of whether that gap is increasing or decreasing in length between events. Given the high rate of events and observational data our ability to observe and identify repeat events provides a large cohort on which to model the event patterns. We have used data from an administrative database which, while providing a census of HF hospitalizations in the province of Alberta, does not contain other important indicators of clinical significance. Additional data such as ejection fraction or degree of cardiac remodeling may improve the prediction of our models.

One limitation of the multistate methods is that the outcome states need to be defined and each additional state increases the model complexity exponentially. As such there is still a requirement to create reasonably “simple” groupings and it is difficult to consider every possibility. The multi-state strategy does allow for the important comparison of competing risk conditional on the current state of the patient. For example, a patient with advanced age has a slightly increased risk of another HF hospitalization but is at much higher risk of death out of hospital; if in hospital, for any reason, the hazard ratio of death is smaller. The reasons for the differences in hazard ratio may lie in which patients are healthy enough to be re-hospitalized, are more aware of their condition and seek treatment sooner or some other feature worthy of further investigation. Additionally it is important to consider the impact of each state, a patient in hospital can be discharged home (another transient state) or have a death even in hospital (an “absorbing” state, meaning that the patient does not leave). The inclusion of a treatment strategy could be included as a factor in the model which would allow the identification of which transitions are impacted and to what degree. This type of model could form the foundation for a Markov model (which typically requires the transition probabilities to be pre-specified rather than estimated) and may be clinically useful if deployed in an electronic health record in order to deploy appropriate resources for individual patients flagged as being at above average risk for adverse outcomes. For example, a targeted outpatient intervention may be possible for patients at a high risk of re-hospitalization. We were unable to include medications in our study but this would be next step if it were to include drug type and dose or other interventions into the model.

The development of multi-state models is complex and would benefit from longer term follow-up data and registry/population level data to populate the transitions between states. In the current era of quality metrics, monitoring of these data is becoming increasingly common.

We described the ‘gap-time’ for patients with heart failure and demonstrated the importance of separating the sources of competing risk for HF patients during follow-up. The gap-time decreased between successive HF hospitalizations, even after accounting for mortality. The use of information such as the gap time provides additional insight into patient experiences (and a potential other outcome for comparative effectiveness studies) beyond the occurrence of short term composite endpoints or time to first even analyses, and is particularly important for chronic diseases like heart failure.
